# Impact of Individual Characteristics on Hospital Outcomes in Exacerbated COPD in a Biomass-Exposed Turkish Population

**DOI:** 10.3390/jcm13226838

**Published:** 2024-11-14

**Authors:** Fatih Uzer, Burcu Karaboğa, Aliye Gamze Calis, Nermin Kaplan, Emsal Sema Altınöz, Sena Sahin, Mustafa Karaca

**Affiliations:** 1 Department of Respiratory Diseases, Akdeniz University, 07070 Antalya, Türkiye; md.fuzer@gmail.com (F.U.); semaltı; 2 Department of Respiratory Diseases, Antalya Ataturk State Hospital, 07070 Antalya, Türkiye; 3 Department of Respiratory Diseases, University of Health Sciences Antalya Training and Research Hospital, 07070 Antalya, Türkiye; 4 Department of Medical Oncology, Akdeniz University, 07070 Antalya, Türkiye

**Keywords:** smoking status, hospital outcomes, COPD, exacerbations

## Abstract

**Background**: Chronic obstructive pulmonary disease (COPD) is a leading cause of morbidity and mortality globally, and factors such as biomass exposure, demographic characteristics, and comorbidities significantly influence patient outcomes during exacerbations. **Aim**: This study aims to clarify the impact of patient characteristics on key hospital outcomes, including ICU admissions, hospital length of stay, and in-hospital mortality, focusing on the contextual role of biomass exposure rather than its direct impact. **Methods**: Using a multicenter, retrospective cohort design, we analyzed the medical records of patients admitted with COPD exacerbations from January 2021 to December 2023. Eligible patients were over 40 years old with confirmed COPD exacerbation, excluding those with other significant lung conditions, severe organ dysfunction, or incomplete data. The collected data included demographics, smoking history, comorbidities, medications, laboratory results, and clinical outcomes, with smoking status categorized into current, former, or never smokers. **Results**: Our analysis comprised 334 patients with a mean age of 69 ± 8.8 years, including 52 (15.6%) females. Biomass exposure, observed in 22% of patients, was associated with a higher likelihood of being female (*p* < 0.001), lower smoking rates (*p* < 0.001), higher prevalence of diabetes mellitus type 2 (*p* = 0.020), lower peripheral blood eosinophilia (*p* = 0.001), increased intensive care unit (ICU) admissions (*p* = 0.034), and higher in-hospital mortality (*p* = 0.043). Non-survivors tended to be older and had a higher prevalence of hypertension, a history of childhood pneumonia, longer COPD duration, greater need for non-invasive ventilation (NIV) during hospitalization, and more frequent ICU admissions. Univariate Cox regression analysis revealed no significant associations between characteristics and outcomes. **Conclusions**: Patients with biomass exposure were more likely to be female and had higher rates of ICU admission and in-hospital mortality.

## 1. Introduction

Exacerbations of chronic obstructive pulmonary disease (COPD), marked by an acute worsening of symptoms, often lead to hospitalization and significantly contribute to the global burden of the disease, with an estimated three million deaths annually attributed to COPD [1]. Although cigarette smoking remains a primary risk factor, recent studies highlight that COPD encompasses diverse clinical presentations affected by varied exposures, such as biomass smoke, air pollution, and occupational hazards, as well as factors such as genetic predisposition and lung development abnormalities. These findings indicate that COPD is a heterogeneous condition, not limited to a single cause, and reinforce the need to consider environmental, developmental, and genetic influences in its taxonomy [2,3,4].

Although tobacco use is a primary factor, additional risks include air pollution, biomass exposure, childhood asthma, pneumonia, advanced age, malnutrition, low socioeconomic status, genetic factors, and occupational exposures [1,5,6,7,8,9]. In Turkey, biomass exposure remains a significant risk factor for COPD, especially in rural areas where traditional heating and cooking methods are still prevalent. Studies show that up to 4.2% of COPD patients have been exposed to biomass [5]. These factors contribute to the disease’s variability, affecting both its progression and the frequency of exacerbations.

The clinical characteristics of stable COPD patients with varying levels of smoking have been addressed in numerous studies [5,10,11,12,13]. However, limited research has addressed the impact of biomass exposure on outcomes following acute COPD exacerbation. This study aimed to clarify the impact of patient characteristics on key hospital outcomes, including ICU admissions, hospital length of stay, and in-hospital mortality, focusing on the contextual role of biomass exposure rather than its direct impact.

## 2. Materials and Methods

Study Design: We used a multicenter, retrospective cohort design to assess the outcomes of patients hospitalized for COPD exacerbations. Data from the medical records of individuals admitted between 1 January 2021 and 31 December 2023 were analyzed retrospectively. The sample size was determined using the G-power program. The sample size was calculated to achieve a power of 90% with an alpha value of 0.05. According to this, a minimum of 332 patients was required.

Study Population: The study population comprised patients diagnosed with COPD exacerbation who were admitted to Akdeniz University, Antalya Atatürk State Hospital and University of Health Sciences Antalya Training and Research Hospital. The inclusion criteria were individuals aged >40 years who had a confirmed diagnosis of COPD exacerbation, determined by clinical and radiological criteria according to the Global Initiative for Obstructive Lung Disease (GOLD) guidelines [1]. The exclusion criteria were as follows: (1) diagnosed with comorbid lung conditions, such as lung cancer, active pulmonary tuberculosis, pulmonary embolism, or interstitial lung disease; (2) history of prior lung surgery; (3) asthma, asthma–COPD overlap syndrome (ACOS), or severe heart, liver, or kidney dysfunction; (4) septicemia; and (5) missing data. Patients with unavailable pulmonary function test results and smoking history were considered to have missing data and were therefore excluded.

Data Collection: Patient data was retrieved from electronic medical records and included demographic information (age and gender), smoking history, comorbidities, medications, laboratory results, and clinical outcomes. Smoking status was categorized into three groups: current smokers, former smokers, and non-smokers. A current smoker was defined as an individual who currently smokes at least one cigarette a day. A former smoker was defined as someone who had quit smoking for more than 1 year. A never smoker was defined as an individual with a lifetime exposure of fewer than 1 pack of cigarettes per year. The group identified as having biomass exposure consisted of individuals who used wood, animal dung, or coal for cooking or heating purposes. SpO_2_ represents the oxygen saturation measured by a fingertip saturation device in room air, whereas PO_2_ corresponds to the partial pressure of oxygen in arterial blood gas obtained from room air. The level of airway obstruction was categorized according to the GOLD guidelines as stages 1, 2, 3, and 4. The patient’s classification into groups (A, B, and E) was defined based on the GOLD guidelines. Accordingly, all patients were classified as group E according to the GOLD guidelines due to experiencing at least one exacerbation requiring hospitalization. The indications for non-invasive ventilation (NIV) include at least one of the following: respiratory acidosis (PaCO_2_ ≥ 6.0 kPa or 45 mmHg and arterial pH ≤ 7.35), severe dyspnea with clinical signs suggestive of respiratory muscle fatigue or increased work of breathing (such as the use of respiratory accessory muscles, paradoxical motion of the abdomen, or retraction of the intercostal spaces), or persistent hypoxemia despite supplemental oxygen therapy. Patients requiring NIV received treatment during their ward stay, with additional support provided in the ICU when indicated. Admission to the ICU was determined by the attending physician based on clinical judgment.

Outcome Measures: the primary outcome measures were in-hospital mortality and the requirement for intensive care, whereas the secondary outcomes included hospital length of stay (LOS) and intensive care unit admission.

Statistical Analysis: Statistical analyses were conducted using SPSS 28.0 to evaluate hospital outcomes among current smokers, former smokers, and non-smokers, as well as between survivors and non-survivors, and patients with and without biomass exposure. Descriptive statistics for continuous variables were presented as mean ± standard deviation or median, whereas categorical variables were expressed as frequencies and percentages. Group comparisons were performed using *t*-tests or analysis of variance (ANOVA) for continuous variables, and chi-square tests for categorical variables. To account for confounding variables and time-to-event outcomes (hospital mortality, ICU admission, and ICU and hospital discharge), Cox regression analysis was applied. The confounders included in these models were selected based on statistical significance in the univariate analysis. A *p*-value < 0.05 was considered statistically significant.

Ethical Considerations: This study adhered to the ethical principles outlined in the Declaration of Helsinki. Ethical approval was obtained from the Institutional Review Board (IRB) of Akdeniz University before commencing data collection (date: 29 February 2024; number: 95).

## 3. Results

**Figure 1:**
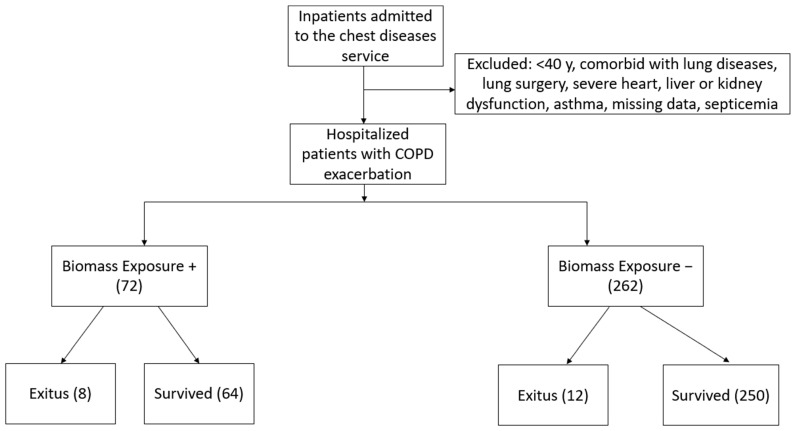
Flow chart of the patients.

This study included 334 patients with a mean age of 69 ± 8.8 years, of whom 52 (15.6%) were female. Among patients hospitalized for COPD exacerbation, 11.2% (*n* = 8) had a history of biomass exposure. The flowchart is presented in Figure 1. Additionally, 32 patients (10%) had a history of tuberculosis, 53 (16%) had childhood pneumonia, and 53 (16%) had childhood asthma. Regarding smoking status, 172 patients (51%) were current smokers, 126 (38%) were former smokers, and 36 (11%) were never smokers. The most frequently identified comorbidities were hypertension (*n* = 134), coronary artery disease (*n* = 82), and diabetes mellitus type 2 (*n* = 74). Patients with COPD who were exposed to biomass were more likely to be female (*p* < 0.001), less likely to be smokers (*p* < 0.001), had a higher prevalence of diabetes mellitus (*p* = 0.020), and showed lower levels of peripheral blood eosinophilia (*p* = 0.001). Additionally, those with biomass exposure had a higher rate of ICU admissions (*p* = 0.034) and increased in-hospital mortality (*p* = 0.043) (Table 1).

**Figure 2:**
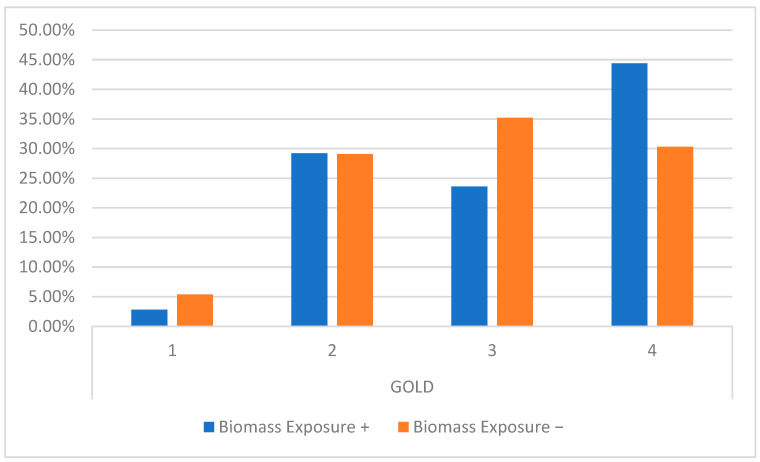
Distribution of obstruction levels (GOLD 1, 2, 3, and 4) among the groups.

There were no differences among the groups in the degree of airway obstruction (GOLD 1, 2, 3, and 4) (Figure 2).

A comparison of patients based on smoking status is provided in Table 2. The clinical characteristics that differed among the three groups were gender, history of diabetes mellitus type 2, history of tuberculosis, history of childhood pneumonia, history of asthma, biomass exposure, cough, duration of intensive care stay, COPD exacerbations in the previous year, Modified Medical Research Council (MMRC) score, and forced expiratory volume in 1 s (FEV1). Never smokers had longer hospital and ICU stays, as well as longer durations of NIV use during hospitalization, compared to the other two groups. Long-term oxygen therapy (LTOT) use was more common among current smokers.

The non-survivors were older and had a higher prevalence of hypertension, a higher prevalence of childhood pneumonia, a longer duration of COPD, a greater need for NIV during hospitalization, and a higher rate of ICU admission. In addition, non-survivors had a lower resting fingertip oxygen saturation and arterial oxygen levels while breathing room air (Table 3).

The univariate Cox regression analysis showed no significant association between any of the characteristics and any of the outcomes. However, we chose to include fingertip saturation and body mass index (BMI) as covariates based on their previous association with COPD and exacerbations [14,15]. The Cox regression analysis showed that smoking status was not associated with time to death in the hospital. Similarly, there were no significant associations between any of the independent variables, including smoking status, and ICU admission, ICU length of stay, or hospital LOS.

## 4. Discussion

The present study aimed to assess the outcomes of hospitalized COPD exacerbation patients in relation to their biomass exposure, comparing those with and without a history of biomass exposure. We observed a notable prevalence of comorbidities, early-life respiratory conditions (asthma and pneumonia), and biomass exposure among non-smoking COPD patients, highlighting non-tobacco-related risk factors that contribute to disease progression. Patients with biomass exposure were more likely to be female and had higher rates of ICU admission and in-hospital mortality. Additionally, current smokers showed a higher rate of home NIV use, suggesting more advanced disease and increased respiratory failure.

Chronic obstructive pulmonary disease, a widespread and debilitating respiratory condition with persistent airflow limitation, affects millions worldwide and is among the leading causes of death [1]. Although tobacco use is a primary factor, additional risks include air pollution, biomass exposure, childhood asthma, pneumonia, advanced age, malnutrition, low socioeconomic status, genetic factors, and occupational exposures [1,5,6,7,8,9,16]. Global data on indoor and outdoor pollutants, including insights from forest fire studies, reinforce the link between smoke exposure and heightened COPD morbidity, highlighting the importance of reducing biomass use, particularly in resource-limited rural areas [9,17]. COPD patients exposed to biomass smoke experience exacerbation risks comparable to those exposed to tobacco, underscoring the need for active treatment and comprehensive management, including ventilation improvements and alternative fuel sources [18]. In our study, patients with biomass exposure had higher ICU admission rates and increased mortality; however, these differences were not statistically significant in the regression analysis.

The present study showed a higher prevalence of never-smoker COPD patients among females, consistent with previous studies that have suggested gender-based differences in COPD manifestation. Similar to our study, Bajpai et al. [11] found a preponderance of female never-smoker patients among stable COPD cases. This demographic trend is consistent with other studies [2,5,12,19,20] and extends to COPD exacerbations, where a significant portion of never-smoker patients, particularly females, has been observed [21,22,23]. This underscores the importance of understanding and addressing COPD in never smokers, given its complex interaction with various risk factors.

As expected, COPD patients, often older and predominantly smokers, exhibit a high prevalence of comorbidities [5,23]. In our study, we observed a notable prevalence of comorbidities, such as diabetes mellitus type 2, hypertension, and coronary artery disease, which aligns with previous findings in COPD populations. Of particular interest, we found higher rates of childhood asthma and pneumonia among non-smokers, suggesting that early-life respiratory events may contribute to COPD development in this group. Additionally, biomass exposure, particularly in non-smokers, was another significant factor, reflecting the impact of environmental exposures on respiratory health in Turkey. These findings are consistent with previous studies that highlight the role of non-tobacco-related risk factors in COPD progression. Notably, the 27.8% incidence of a history of tuberculosis in never-smoker patients exceeds previous reports, providing insights into historical trends [15,24]. This multifaceted association underscores the need for comprehensive patient management, recognizing diverse health conditions that may influence COPD outcomes. Furthermore, the increased utilization of home NIV in current smokers points to more advanced disease or higher rates of respiratory failure in this subgroup, possibly due to the continued harmful effects of smoking. These factors, including comorbid conditions and environmental exposures, underscore the complex, multifactorial nature of COPD and its exacerbations, particularly in non-smoking populations.

The in-hospital mortality of COPD varies between 12% and 29% [25]. The data regarding long-term mortality in COPD patients, both with and without a history of smoking, are conflicting. In a study conducted in Mexico [26], it was reported that patients with COPD who were ever smokers had a higher mortality rate compared to those with COPD attributed to biomass exposure, observed over a 7-year follow-up period. In a study conducted by Thomsen and colleagues [27], a higher incidence of cardiac death events was observed in stable COPD patients who were current or former smokers. However, a similar study conducted by Josephs and his colleagues [28] showed that, over the course of 3 years, a significantly greater proportion of former smokers and never smokers died compared to current smokers. Despite our best efforts, we could not find any previously published data regarding the relationship between smoking status, biomass exposure, and hospital mortality in patients with COPD exacerbations. In stable COPD studies, the requirement for ICU admission is lower, whereas studies involving hospitalized exacerbation cases suggest a higher rate of ICU admissions. After adjusting for BMI and fingertip oxygen saturation on admission, we found no association between biomass exposure and hospital mortality.

In patients hospitalized with COPD, the need for intensive care ranges from 2% to 19% [25]. Increased BUN, low SpO_2_, advanced age, leukopenia, low FEV1 (<50%), heart failure, and an elevated eosinophil–neutrophil ratio are associated with intensive care admission [25]. In both univariate and multivariate regression analyses, we determined that biomass exposure is not associated with ICU admission. Although the univariate analysis showed that patients with biomass exposure had a higher ICU admission rate and increased in-hospital mortality, the adjusted multivariate analysis showed no association between biomass exposure and either ICU admission rate or in-hospital mortality. This finding could be explained by the heterogeneous nature of COPD or may be related to the substantial proportion of patients in our study classified as GOLD E. Patients in Turkey with COPD who are exposed to biomass tend to be predominantly female, have a higher prevalence of diabetes mellitus type 2, and lower levels of peripheral blood eosinophilia, all of which may contribute to their increased rates of ICU admissions and higher in-hospital mortality, suggesting that the demographic and comorbidity profile of these patients exacerbates the severity of their condition.

In a study of patients who had ischemic stroke, COPD, and lung cancer, it was reported that patients without a smoking history had shorter hospital stays compared to those with a smoking history. However, there were no subgroup analyses by diagnosis or the severity of illness [29]. We found no association between smoking status and hospital LOS.

Despite the valuable insights gained from this study, it is crucial to acknowledge its limitations. The retrospective design introduces inherent biases, and the sample size might limit the generalizability of the findings. ICU admission was based on clinical judgment, which may be considered a limitation. Another limitation could be the relatively small number of patients with mortal outcomes in our study. The strengths of this study include its relatively large sample size and the use of multivariate analyses.

## 5. Conclusions

This study provides novel insights into the outcomes of COPD patients exposed to biomass, particularly highlighting their demographic profile and health complications. Our findings reveal that patients with biomass exposure are predominantly female and experience higher rates of ICU admissions and in-hospital mortality compared to their counterparts without such exposure. Furthermore, the increased utilization of home non-invasive ventilation (NIV) among current smokers suggests a more advanced stage of disease and a greater risk of respiratory failure in this subgroup.

These results underscore the importance of recognizing non-tobacco-related risk factors in COPD management, particularly in populations exposed to biomass. Understanding the unique characteristics and challenges faced by this demographic can help tailor interventions to improve patient outcomes. Future research should focus on the long-term implications of biomass exposure on COPD progression and outcomes, as well as strategies for effective management in these high-risk populations. By addressing these issues, we can enhance our understanding of COPD and improve care for affected individuals worldwide.

## Figures and Tables

**Table 1:** Comparison of clinical characteristics of COPD patients with and without biomass exposure.

	Biomass Exposure + (*n* = 72)	Biomass Exposure − (*n* = 262)	Total (%)	*p*-Value
Age (years) (mean ± sd)	68.5 ± 8.1	69.0 ± 9.0	68.9 ± 8.8	0.688
Female, *n* (%)	22 (30.6)	30 (11.5)	52 (15.6)	<0.001
BMI (kg/m^2^) (mean ± sd)	24.1 ± 5.6	24.7 ± 5.2	24.6 ± 5.3	0.347
Current smoker, *n* (%)	25 (34.7)	147 (56.1)	172 (51.7)	<0.001
Former smoker, *n* (%)	23 (31.9)	103 (39.3)	126 (37.5)	
Never smoker, *n* (%)	24 (33.3)	12 (4.6)	36 (10.8)	
Hypertension, *n* (%)	32 (44.4)	102 (38.9)	134 (40.1)	0.238
Diabetes Mellitus type 2, *n* (%)	23 (31.9)	51 (19.5)	74 (22.2)	0.020
CAD, *n* (%)	22 (30.6)	60 (22.9)	82 (24.6)	0.120
LTOT, *n* (%)	31 (43.1)	100 (38.2)	131 (39.2)	0.268
NIV (during hospital), *n* (%)	26 (36.1)	76 (29)	102 (30.6)	0.404
ICU need, *n* (%)	22 (30.6)	51 (19.5)	73 (21.9)	0.034
Mortality, *n* (%)	8 (11.1)	12 (4.6)	20 (6)	0.043
Smoking (pack years)	43.1 ± 18.1	42.6 ± 16.5	52.7 ± 26.4	0.129
Duration of COPD (years)	11.6 ± 7.8	8.9 ± 6.9	9.7 ± 7.2	0.09
MMRC	3.1 ± 1.1	3.0 ± 0.9	3.0 ± 0.9	0.753
FEV 1 (%)	61.6 ± 10.2	59.6 ± 16.8	1.2 ± 0.5	0.847
COPD exacerbation (previous year), *n* (%)	54 (75)	201 (76.7)	255 (76.3)	0.817
Number of COPD exacerbations (previous year)	2.7 ± 2.3	1.9 ± 1.6	2.1 ± 1.7	0.002
CRP (mg/dL)	79.8 ± 81.6	95.4 ± 113.3	88.1 ± 102.7	0.348
Eosinophil (Cell/mcL)	49.2 ± 72.1	126.1 ± 329.6	109.4 ± 295.8	0.001
SPO_2_	84.4 ± 6.9	86.1 ± 7.1	85.7 ± 7.1	0.081
PO_2_ (mmHg)	54.8 ± 9.1	57.7 ± 14.4	57.7 ± 13.1	0.062
pCO_2_ (mmHg)	45.1 ± 17.1	44.6 ± 14.2	44.7 ± 15.0	0.811
pH	7.38 ± 0.1	7.38 ± 0.1	7.38 ± 0.1	0.824
Duration of NIV (during hospitalization) (days)	9.0 ± 10.6	8.1 ± 6.6	8.3 ± 7.9	0.683
LOS (hospital) (days)	8.7 ± 7.2	9.3 ± 6.8	9.2 ± 6.9	0.517

All continuous variables are reported as mean ± SD, and categorical variables are reported as count (percentage). BMI: body mass index, CAD: Coronary artery disease, LTOT: long-term oxygen treatment, NIV: non-invasive ventilation, ICU: intensive care unit, MMRC: Modified Medical Research Council, FEV1: forced expiratory volume in the first second, LOS: length of stay, ICS: inhaled corticosteroid, COPD: chronic obstructive pulmonary disease, CRP: C-reactive protein.

**Table 2:** Comparison of patients based on smoking status.

	Current Smoker (*n* = 172)	Former Smoker (*n* = 126)	Never Smoker (*n* = 36)	*p* Value
Gender, female, *n* (%)	12 (7.0)	15 (11.9)	25 (69.4)	<0.001
Age (years)	71.3 ± 7.9	64.6 ± 8.1	72.6 ± 9.0	0.818
BMI (kg/m^2^)	24.9 ± 5.7	24.2 ± 4.7	24.6 ± 5.0	0.114
Coronary artery disease, *n* (%)	54 (31.4)	20 (15.9)	8 (22.2)	0.008
Hypertension, *n* (%)	73 (42.4)	45 (35.7)	16 (44.4)	0.431
Diabetes Mellitus type 2, *n* (%)	44 (25.6)	18 (14.3)	12 (33.3)	0.016
Tuberculosis, *n* (%)	15 (8.7)	7 (5.6)	10 (27.8)	<0.001
Childhood pneumonia, *n* (%)	15 (8.7)	21 (16.7)	17 (47.2)	<0.001
Childhood asthma, *n* (%)	19 (11.0)	16 (12.7)	18 (50.0)	<0.001
Biomass exposure, *n* (%)	25 (14.5)	23 (18.3)	24 (66.7)	<0.001
Cough, *n* (%)	78 (45.3)	72 (57.1)	23 (63.9)	0.040
Dyspnea, *n* (%)	158 (91.9)	111 (88.1)	29 (80.6)	0.121
Sputum, *n* (%)	54 (31.4)	46 (36.5)	18 (50.0)	0.099
GOLD (4)	61 (35.5)	43 (34.1)	7 (19.4)	0.327
COPD exacerbation (previous year), *n* (%)	141 (82.0)	92 (73.0)	22 (61.1)	0.041
SpO_2_ (%)	85.9 ± 6.7	85.3 ± 7.1	86.2 ± 7.0	0.854
PO_2_ (mmHg)	58.1 ± 14.1	57.2 ± 11.0	57.7 ± 15.9	0.891
PCO_2_ (mmHg)	45.2 ± 13.8	44.6 ± 13.8	43.9 ± 16.7	0.908
pH	7.38 ± 0.1	7.38 ± 0.1	7.38 ± 0.1	0.902
MMRC	3.2 ± 0.9	2.9 ± 0.9	2.6 ± 1.2	0.003
FEV 1 (lt)	1.15 ± 0.5	1.31 ± 06	1.13 ± 0.5	0.027
Duration of COPD (years)	11.1 ± 7.5	8.6 ± 6.5	7.9 ± 7.3	0.425
LOS (hospital) (days)	9.3 ± 6.9	8.7 ± 6.1	10.1 ± 8.9	0.175
LOS (ICU) (days)	3.3 ± 4.6	2.2 ± 3.7	6.0 ± 8.2	0.020
ICS use, *n* (%)	146 (84.9)	108 (85.7)	28 (77.8)	0.497
LTOT, *n* (%)	82 (47.7)	37 (29.4)	12 (33.3)	0.004
Intensive care unit, *n* (%)	40 (23.3)	22 (17.5)	11 (30.6)	0.200
NIV (at home), *n* (%)	45 (26.2)	8 (6.3)	4 (11.1)	<0.001
Duration of NIV (during hospitalization)	7.9 ± 7.2	6.9 ± 4.0	13.8 ± 14.1	0.030
Mortality, *n* (%)	12 (7.0)	4 (3.2)	4 (11.1)	0.153

All continuous variables are reported as mean ± SD, and categorical variables are reported as count (percentage). BMI: body mass index, GOLD: Global Initiative for Obstructive Lung Disease, COPD: chronic obstructive pulmonary disease, MMRC: Modified Medical Research Council, FEV1: forced expiratory volume in the first second, LOS: length of stay, ICS: inhaled corticosteroid, LTOT: long-term oxygen treatment, NIV: non-invasive ventilation.

**Table 3:** Comparison of clinical characteristics of non-survivors and survivors.

	Non-Survivors (*n* = 20)	Survivors (*n* = 314)	*p*-Value
Age (years)	74.1 ± 8.7	68.6 ± 8.7	0.007
Female, *n* (%)	6 (30)	46 (14.6)	0.07
BMI (kg/m^2^)	22.4 ± 5.3	24.7 ± 5.3	0.07
Current smoker, *n* (%)	12 (60)	160 (51.0)	0.15
Former smoker, *n* (%)	4 (20)	122 (38.9)	
Never smoker, *n* (%)	4 (20)	32 (10.2)	
Hypertension, *n* (%)	14 (70)	120 (38.2)	0.005
Diabetes Mellitus type 2, *n* (%)	8 (40)	66 (21)	0.05
Childhood pneumonia, *n* (%)	9 (45)	44 (14)	0.001
Biomass exposure, *n* (%)	8 (40)	64 (20.4)	0.04
LTOT, *n* (%)	12 (60)	119 (37.9)	0.05
NIV (during hospital), *n* (%)	15 (75)	87 (27.7)	<0.001
ICU need, *n* (%)	19 (95)	54 (17.2)	<0.001
Smoking (pack years)	49.1 ± 15.2	52.9 ± 26.8	0.58
Duration of COPD (years)	13.6 ± 9.6	9.4 ± 7.0	0.03
MMRC	3.4 ± 0.9	3.0 ± 0.9	0.06
FEV 1 (%)	39.4 ± 15.6	42.9 ± 16.8	0.36
Number of COPD exacerbations (previous year)	2.4 ± 1.8	2.0 ± 1.8	0.40
CRP (mg/dL)	119 ± 93.2	89.3 ± 106.7	0.31
Eosinophil (Cell/mcL)	66.5 ± 93.2	112.3 ± 303.7	0.50
SpO_2_ (%)	82.1 ± 8.1	85.9 ± 7.0	0.03
PO_2_ (mmHg)	50.1 ± 7.5	57.4 ± 13.4	0.03
PCO_2_ (mmHg)	45.4 ± 14.9	44.7 ± 15.1	0.865
pH	7.37 ± 0.1	7.38 ± 0.1	0.805
Duration of NIV (during hospitalization) (days)	8.9 ± 11.3	8.3 ± 7.2	0.856
LOS (hospital) (days)	11.6 ± 10	9.1 ± 6.6	0.11

All continuous variables are reported as mean ± SD, and categorical variables are reported as count (percentage). BMI: body mass index, LTOT: long-term oxygen treatment, NIV: non-invasive ventilation, ICU: intensive care unit, MMRC: Modified Medical Research Council, FEV1: forced expiratory volume in the first second, LOS: length of stay, ICS: inhaled corticosteroid, CRP: C-reactive protein.

## Data Availability

The data is unavailable due to privacy and ethical restrictions.
